# Does the Price Support Policy Drive a Balanced Distribution of Profits in the Chinese Dairy Supply Chain? Implications for Supplier and Consumer Psychology

**DOI:** 10.3389/fpsyg.2021.632355

**Published:** 2021-03-01

**Authors:** Feng Hu, Xun Xi, Rongjian Yu, Rong Xiang, Yueyue Zhang, Zhimin Ren, Xiaoping Wang, Jie Xie

**Affiliations:** ^1^Global Value Chain Research Center, Zhejiang Gongshang University, Hangzhou, China; ^2^Zhejiang Postdoctoral Station, NingboTech University, Ningbo, China; ^3^School of Economics, Zhejiang Gongshang University, Hangzhou, China

**Keywords:** dairy supply chain, price policy effect, profit balance, multi-period DID, supplier psychology, consumer psychology

## Abstract

Evaluating the price policy of raw milk is of great significance to the sustainable development of an industry supply chain. In this context, our study used the multi-period difference-in-difference method to systematically examine the impact of the policy implementation on product price and profit distribution in the supply chain. The results showed the following: (1) the price of raw milk in the implementation area of the price support policy is 13.54% higher than that of the unimplemented area; (2) the effect of price increase in the western region (15.5%) is higher than that in the eastern region (13%), and the central region (10.73%); (3) furthermore, the purchase price guidance policy of raw milk drives price increase or price suppression in the links of the supply chain to promote a balanced distribution of profits among the participants in the chain. These conclusions all have good stability and have reference significance for further improving and adjusting the price support policy of raw milk to realize the sustainable development of the Chinese dairy industry. This will enhance the production confidence of Chinese raw milk producers and improve Chinese consumers’ expectations and consumer psychology regarding domestic dairy products.

## Introduction

The dairy supply chain includes multiple links, such as forage cultivating and processing, dairy animal feeding, dairy processing, and sales. Therefore, promoting the sound development of the dairy industry is beneficial to realizing income increase for dairy farmers, improving the dietary structure of residents, driving the development of enterprises, and local economy (Tinbergen, 1964; Schmitz and Helmberger, 1970). After 10 years of reflection and adjustment, the development mode and product quality of the Chinese dairy industry has improved significantly. For example, the casual inspection pass rate of raw milk in 2018 was 99.8%, the melamine index has been 100% qualified for 10 successive years^1^. In 2018, the number of cows reached 14,300,000, and the annual single yield was over 6,350 kg, which was 1.5 times of that of 2012^2^; and in the period of 2012--2018, the total value of output of dairy enterprises above the state-designated scale increased from 246.9 billion yuan to 309.5 billion yuan, realizing successive growth for 5 years^3^. However, it is undeniable that the profit distribution in the whole dairy supply chain in China is imbalanced, which means that the profit of dairy retailers and dairy processing enterprises is far greater than the profit of raw milk suppliers. Under the influence of dual price transfer from downstream dairy enterprises and retailers to dairy farmers, the raw milk selling price is always being squeezed. This imbalance has been widely recognized by scholars in the field.

Based on the provincial research data of Heilongjiang Province, Qian et al. (2011) analyzed the costs and profits of each link, including production by dairy farmers, processing by dairy enterprises, and retail by supermarkets, and the results have shown that the profit added value of dairy products is mostly distributed in the retail link, which is followed by the finished dairy product processing link, while the raw milk production link has the least profit added value, which suggests that the profit distribution in the dairy supply chain is arbitrary. Zhong et al. (2014) discussed the differentiation of profit among the subjects of the dairy industry from the perspectives of production costs, and the results have shown that the ratio of dairy cow feeding cost, product processing cost, and selling cost is approximately 7.5: 1.5: 1 and the dairy farmers are facing higher production risks than dairy enterprises and retailers. Hu et al. (2020) discussed the market price transmission mechanism from the perspective of a reorganization of the dairy supply chain, and by adopting the impulse response function and error correction method, demonstrated that there is asymmetric price transmission among the production links of the dairy supply chain. This means that the probability of cost shift from production link to retail link is far lower than that from retail link to production link. In addition, other studies on the current status of profit distribution in the dairy supply chain have also proved that there is an imbalanced profit distribution in the chain.

A balanced profit distribution will bring more economic benefits to producers. When production costs remain the same, producers will be more able to resist external risks and more confident in increasing production and R&D investment while improving product quality (Bryant and Dillard, 2019; Dutta et al., 2019). For consumers, the enrichment of product categories and the improvement of product quality will strengthen their confidence in domestic products (Carmen et al., 2016; Jimenez-Delgado and Reina-Paz, 2020). For both producers and consumers, their increased psychological expectations will be more conducive to the development of the dairy industry and price stability, thus forming a virtuous cycle (Silva et al., 2017; Kniffin et al., 2018; Dutta et al., 2019).

The import flow of raw milk in China has been increasing annually, and the production profits of dairy farmers have been “double-squeezed” by dairy companies and retailers, which has severely affected the enthusiasm of dairy farmers in production. The fundamental goal of the price support policy is to solve the problem of unfair transactions in the raw milk market and increase the production confidence of dairy farmers. Furthermore, the increase in producer confidence is conducive to the improvement of product quality, which helps consumers overcome psychological barriers in resisting domestic dairy products and stimulating their consumption vitality.

The balanced profit distribution among the production links of the dairy supply chain is vital for the sustainable development of the industrial development mode (Alvarez and Arias, 2004; Hu et al., 2019). This is because a balanced profit distribution among the production links stimulates the vitality of producers and the potential of consumers, making the producer prefer to emphasize quality over quantity of products, and making the consumers prefer domestic dairy products, thus forming a sustainable virtuous cycle. To further correct the market inequalities in the industry and promote a balanced profit distribution, the state governments issued *Opinions of the State Council on Promoting the Sustainable and Healthy Development of the Dairy Industry* (hereinafter, referred to as “*Opinions*”), *Dairy Products Quality and Safety Supervision and Administration Regulations* (hereinafter, referred to as “*Regulations*”) in 2007 and 2008, respectively, according to which the local governments shall formulate raw milk purchase guide prices in case of raw milk market failure, and timely implementation of a guide price policy in the areas in which the purchase price of raw milk is relatively low. The purpose of such a policy is to protect the minimum guaranteed profit of farmers and fundamentally eliminate imbalance in the purchase and selling price of raw milk. However, there is no literature verifying the actual effect of raw milk price support policy. In fact, the effects of the raw milk price support policy should be systematically assessed for policy adjustment and for bridging the mentioned theoretical gap. For these reasons, this paper discusses the actual effect of the price support policy on subjects in the dairy supply chain, aiming to answer the following questions: whether the raw milk purchase guide price policy drives an increase in the average selling price of such products, and whether it promotes balanced profit distribution among the subjects in the dairy supply chain.

Since the issuance of *Opinions* and *Regulations*, 19 provinces and municipalities directly under the central government have issued raw milk purchase guide price policies, with the purpose of improving raw milk price management measures, standardizing product transactions, and therefore eliminating the unfairness in the purchase and selling price of the dairy supply chain. As shown in Table 1, such policies were implemented at different dates in these provinces. For example, Hebei, Shanxi, and Shaanxi issued related local industry policies such as *Agricultural-related Price (Charge) Policy in Hebei Province, Opinions of the People’s Government of Shaanxi Province on the Implementation of the “Regulations on the Supervision and Administration of Milk Quality and Safety”* immediately after the issuance of the regulations; while provinces such as Jiangsu, Jiangxi, and Qinghai issued the raw milk purchase guide price policy around 2018, lagging behind Hebei and other provinces; the reason for this is that the issuance of a policy may be affected by two factors. On the one hand, the production cost and selling price of raw milk vary from area to area, resulting in differences in benefit coordination among subjects of the dairy supply chain; on the other hand, under the effect of the orientation of industry, market, and policy, the dairy industry is not given priority for development in some areas. That is to say, issues such as imbalanced profit distribution among subjects of the dairy industry and imbalanced development in the supply chain will not become the focus of policy in the short term.

**TABLE 1:** Price support policy formulation and policy launch time by province.

Area	Province	Policy proposal time	Related content
Eastern region	Hebei	November 13, 2008	“…calculate the price of raw milk, based on which, propose and publish the raw milk purchase guide price for the peak season (May–October), off season (November to April of following year), and the cases that the price of principal elements affecting raw milk fluctuate significantly.”
	Guangdong	November 26, 2009	“…Guangdong Province Department of Agriculture, the Bureau of Industry and Commerce, the Price Bureau, and Guangdong Dairy Association jointly issued a guide price policy for raw milk purchases. The purchase price per kilogram of milk is 3.83¥(+- 10%). The guide price is expected to be officially implemented in early December. It is reported that this is the first time that Guangdong Province has unified the purchase price of raw milk”
	Fujian	September 30, 2010	“…popularize and promote the *Dairy Farm Hygiene Regulations* and *Raw Milk Production Technical Regulations (Trial)*, and strengthen the guidance and supervision of the local establishment of raw milk price coordination mechanisms.”
	Shanghai	January 16, 2011	Due to the large content of relevant policy documents, we will not list them one by one here.
	Zhejiang	December 29, 2011	
	Shandong	January 29, 2015	
	Beijing	January 3, 2016	
	Jiangsu	November 16, 2018	
Central region	Shanxi	December 18, 2008	
	Hubei	November 27, 2009	
	Heilongjiang	January 16, 2010	
	Henan	May 24, 2012	
	Jiangxi	December 18, 2018	
Western region	Shaanxi	December 18, 2008	
	Xinjiang	January 22, 2009	
	Sichuan	May 14, 2009	
	Ningxia	July 9, 2009	
	Neimenggu	December 27, 2012	
	Qinghai	February 16, 2015	

*The policy launch time is based on the information published on the official websites of the provinces and municipalities. For example, Shandong raw milk purchase price policy information comes from the Departmental Regulatory Documents published by Shandong Provincial People’s Government Official Website (http://www.shandong.gov.cn/).*

**FIGURE 1:**
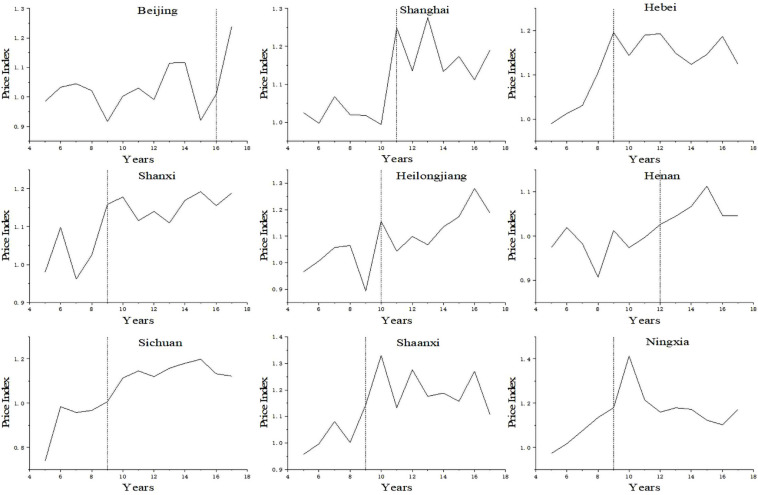
Raw milk purchase price index trends in regions that implemented the policy.

In general, after the implementation of the raw milk purchase guide price policy, the selling price of raw milk and dairy products in the 19 provinces, and in the municipalities directly under the central government showed an upward trend to a varying extent, as shown in Figure 1. In the figure, with three provinces randomly selected from the eastern region, central region, and western region for the policy effect test, the raw milk purchase price index of nine areas such as Beijing, Shanxi, and Sichuan showed an obvious upward trend in the right side of the dividing vertical line (representing the moment of the policy implementation). For example, the raw milk selling price index in Shanghai increased from 0.994 in 2010 to 1.248 in 2011; the raw milk selling price index in Heilongjiang increased from 0.894 in 2009 to 1.155 in 2010; the raw milk selling price index in Ningxia increased from 1.179 in 2009 to 1.412 in 2010. It should be noted that the raw milk purchase guide price policy was issued in 2008 in the Hebei Province, but the raw milk price showed an obvious upward trend in 2009. This was because policies are usually issued in November and December in Hebei, Shanxi, and other areas, and it takes a certain time to implement them in organizations at the municipal and county level. Thus, the policy effect will be shown in the following year. The above-mentioned preliminary test seems to suggest a synchronization between the issuance of policies and the rise of raw milk purchase price, but it cannot demonstrate that the rising price is not the result of other factors. Furthermore, even if the policies have indeed caused an increase in the price, the actual effect should be assessed.

## Materials and Methods

### Model Construction

Is the policy effective? In his study on the influences of post-school education on resident incomes, Ashenfelter (1978) mentioned that temporal and regional characteristics and other random interference factors should be considered to analyze the difference caused by the implementation of a policy. This is because some characteristics of the same area may vary around the time when a policy is implemented, and some characteristics of the area in which the policy is implemented may differ from those of other areas (in which the policy is not implemented). This means that, to assess the actual effect of a policy, it is necessary to exclude other factors that influence the object analyzed as far as possible. Like Ashenfelter, we adopted the difference-in-difference (DID) method, which has been widely applied to evaluate policy effects (Abadie, 2005; Grafova et al., 2014; Wijaya et al., 2020). The expression of the method is shown in Formula (1):

(1) $$Y_{i\,t}=C+\alpha \,P\,e\,r\,i\,o\,d_{t}+\beta \,T\,r\,e\,a\,t\,e\,d_{i}+\gamma \,\left(P\,e\,r\,i\,o\,d_{t}\times T\,r\,e\,a\,t\,e\,d_{i}\right)+Y_{i\,t}=C+\alpha \,P\,e\,r\,i\,o\,d_{t}+\beta \,T\,r\,e\,a\,t\,e\,d_{i}+\gamma \,\left(P\,e\,r\,i\,o\,d_{t}\times T\,r\,e\,a\,t\,e\,d_{i}\right)+\delta \,X+\mu_{i\,t}\delta \,X+\mu_{i\,t}$$

Period_t_ refers to the policy implementation dummy variable, which should be assigned 0 before policy implementation, and 1 after policy implementation. Treated_i_ refers to the grouping dummy variable, which will be assigned 1 if area i is a policy implementation area or organization, that is, in the experimental group, or assigned 0 if the area i is an unimplemented area or organization, that is, in the control group. Period_t_ × Treated_i_ refers to the dummy interaction variable, and its coefficient γ refers to the policy net effect (for specific reasons, please refer to Ashenfelter’s relevant research results, which will not be illustrated here). Finally, X refers to the control variable group and μ_it_ refers to the random error term.

The raw milk purchase guide price policy in China has two characteristics: first, it is a local policy formulated by each province and municipality according to the actual situation and based on the overall institutional policy issued by the central government. Of course, its implementation is not necessary or urgent for areas in which the profit distribution among subjects of the dairy supply chain is relatively balanced; thus, as shown in Table 1, it has not been issued in some areas, providing a control group for continuous reference. Second, local raw milk purchase guide price policies were issued at different times, from 2008 to 2018, which means that the experimental group and control group show a dynamic variation. The first characteristic indicates that the experimental group and control group can be set up in the test of raw milk purchase guide price policy, and the number of observation periods of the research object is greater than 2, which fits in the DID method. However, it should be noted that the traditional DID method requires a consistent implementation time of policies in the experimental group, while the actual implementation time of policies in the 19 areas considered varies to different extents. Thus, raw milk purchase guide price policies cannot meet the condition fully.

To solve the inconsistency in the implementation time of policies, a multiple periods DID was adopted in this study (Dolin et al., 2020; Zhang et al., 2020; Zhao et al., 2020), in which the grouping is based on the policy implementation time instead of whether the area is ultimately affected by the policy. This means that, even if all provinces and municipalities have implemented the raw milk purchase guide price policy, the areas in which such a policy had not been implemented in the study period can be regarded as the control group. The inclusion of this adjustment in Formula (1) is that the interaction variable of the experimental group is no longer the same. In addition, the value variation among the policy implementation dummy variable, grouping dummy variable, and interaction variable remain the same. Thus, Formula (1) can be adjusted to Formula (2):

(2) $$Y_{i\,t}=C+\gamma \,\left(P\,e\,r\,i\,o\,d_{t}\times T\,r\,e\,a\,t\,e\,d_{i}\right)+\delta \,X+\eta_{t}+\varepsilon_{i}+\mu_{i\,t}$$

The difference between Formula (2) and Formula (1) is that the separate policy implementation dummy variable Period_t_ and grouping dummy variable Treated_i_ were excluded; the time fixed effect η_t_ and regional fixed effect ε_i_ were added to eliminate the time effect affecting the estimation of the coefficient of the interaction variable and the regional factors that do not change over time. In addition, the control variable group was retained in Formula (2) to control time-varying variables with regional characteristics, including per capita GDP, population, and some natural environmental factors of each area (Grafova et al., 2014; Khan et al., 2020). Y_it_ refers to the raw milk selling price index of area i in t (year). The other indicators were as defined in Formula (1).

### Data Description

In addition to Beijing, Shanghai, Hebei, and other areas in which the policies were implemented, the sample areas for this research also included 11 areas in which the policies were not implemented, such as Tianjin, Hainan, Guizhou, and Liaoning. It should be noted that in addition to Hong Kong, Macau, and Taiwan, the Tibet Autonomous Region and Jiangxi were excluded from empirical tests, as no source data of the raw milk selling price index were available for reference; thus, 29 sample areas were left out. In addition, considering data availability and the policy implementation time of each area, the research time series was set as 2005–2017. After determining the areas and time series of study, the following indicator data were collected in this paper: first, we defined the core explanatory variable Period_t_ × Treated_i_ (a dummy variable), which indicates that Province i was an area where the raw milk purchase guide price policy was implemented in year t when Period_t_ × Treated_i_ = 1, and that Province i was not an area where the raw milk purchase guide price policy was implemented in year t if Period_t_ × Treated_i_ = 0. Second, the raw milk selling price index was expressed as the ratio of the raw milk selling price index of each year to the commodity price index of the year, whose data were derived from the ‘‘Price Index’’ Column of the *China Economic and Social Big Data Research Platform*.^4^ The ex-factory price index of the finished dairy product was expressed as the ratio of the ex-factory price index of the finished dairy product to the commodity price index of that year, whose data were derived from the 2005–2017 *China Price Statistics Yearbook*.^5^ The retail price index of dairy products was expressed as the ratio of the retail price index of dairy products to the commodity price index of that year, whose data were derived from the same data source used for the ex-factory price index of finished dairy products. Third, the per capita GDP was expressed as the ratio of per capita GDP of each province or municipality to national per capita GDP of that year. The population was expressed as the ratio of the population size of each area to the national population size of that year, whose data were derived from the General Column and Population Column of the *China Economic and Social Big Data Research Platform*.^6^ The data of temperature and precipitation of each area were derived from statistical data from the 2006--2018 *China Meteorological Yearbook*,^7^ and were expressed as the annual mean temperature and precipitation in the main areas of each province. The descriptive statistics of the indicator data are shown in Table 2.

**TABLE 2:** Variables for descriptive statistics.

Variable name	Obs.	Mean	Standard error	Minimum	Maximum
Raw milk selling price index	377	1.05381	0.09448	0.70828	1.41269
Ex-factory price index of finished milk	377	1.01625	0.03882	0.93372	1.13077
Retail price index of dairy products	377	1.00932	0.04868	0.85814	1.17676
Period_t_ × Treated_i_	377	0.31034	0.46324	0	1
GDP per capita index	377	0.03552	0.02734	0.00290	0.12213
Population index	377	0.03301	0.02026	0.00415	0.08034
Temperature (log)	377	2.58812	0.41707	1.45861	3.23474
Precipitation (log)	377	6.62352	0.65759	4.31615	8.68491

## Results

### Regression Result Analysis

The test results of the overall policy effect are shown in Table 3, in which the time fixed effect, regional fixed effect, weather control variables, and regional (time-varying) control variables were added gradually (see columns 1–5). The reported results show that all coefficient values of the interaction variable were positive and were of significance at the 1% level. Thus, the results obtained with Model 5, the most explanatory model, were regarded as the reference standard. In Model 5, the coefficient value of the interaction variable was 0.1354. This showed that, from a holistic perspective, the implementation of a guide price policy in an area could make the raw milk selling price index increase by 0.1354 units, suggesting that the raw milk selling price of that time was 13.54% higher than that of the previous period. Then, does the effect of guide price policy on product selling price remain the same in different areas?

**TABLE 3:** Influence of the raw milk purchase guide price policy on the product price index.

Model	1	2	3	4	5
Period_t_ × Treated_i_	0.1437*** (0.0084)	0.1438*** (0.0102)	0.1398*** (0.0116)	0.1405*** (0.0117)	0.1354*** (0.0131)
Time fixed effect	No	Yes	Yes	Yes	Yes
Regional fixed effect	No	No	Yes	Yes	Yes
Weather control variables	No	No	No	Yes	Yes
Regional (time-varying) control variables	No	No	No	No	Yes
Obs.	377	377	377	377	377
R2	0.4006	0.4644	0.4645	0.4659	0.4694

*** indicate significance at the 1% levels, respectively, the values in parentheses indicate standard errors. Weather control variables include temperature and precipitation. Regional (time-varying) control variables include per capita GDP and population.

The results shown in Table 4 answer this question. In Table 4, Models 6 and 7 represent the policy effect in the eastern region, Models 8 and 9 represent the policy effect in the central region, and Models 10 and 11 represent the policy effect in the western region. Specifically, the guide price policy implemented in the eastern region increased the local raw milk price by 13%, the guide price policy implemented in the central region increased the local raw milk price by 10.73%, and the guide price policy implemented in the western region increased the local raw milk price by 15.5%. Based on this, we can conclude that this policy has a positive driving effect on the raw milk selling price in the eastern, central, and western regions, and the policy effect in the western region is greater than that in the eastern and central regions. The possible reason is that the western region includes the provinces such as Inner Mongolia and Xinjiang, in which the policy orientation and support are more significant than those in the eastern and central regions, and are set to ensure a stable development of animal husbandry.

**TABLE 4:** Testing under conditions of regional heterogeneity.

Model	6	7	8	9	10	11
Period_t_ × Treated_i_	0.1367*** (0.0193)	0.1300*** (0.0220)	0.1193*** (0.0185)	0.1073*** (0.0202)	0.1532*** (0.0210)	0.1550*** (0.0196)
Time fixed effect	Yes	Yes	Yes	Yes	Yes	Yes
Regional fixed effect	Yes	Yes	Yes	Yes	Yes	Yes
Weather control variables	No	Yes	No	Yes	No	Yes
Regional (time-varying) control variables	No	Yes	No	Yes	No	Yes
Obs.	143	143	91	91	143	143
R2	0.5403	0.5512	0.4250	0.4443	0.5169	0.5335

*** indicate significance at the 1% levels, respectively, the values in parentheses indicate standard errors. Weather control variables include temperature and precipitation. Regional (time-varying) control variables include per capita GDP and population.

### Robustness Test

#### Parallel Trend Test

An important prerequisite for the validity of the DID method is the convergence hypothesis (Bogadóttir, 2020; Khan et al., 2020; Sánchez-López et al., 2020). In our study, this means that, if the raw milk purchase guide price policy was not implemented, the price trend in the province or municipality of the experimental group should have been equal to that of the control group. Considering the multiple execution nodes of the guide price policy, the event analysis method was adopted in this study to conduct a parallel trend test. The formula is as follows:

(3) $$Y_{i\,t}=C+\sum_{\Delta =-12}^{\Delta =8}\gamma^{\prime }\,D_{i,t_{0}+\Delta }+\eta_{t}+\varepsilon_{i}+\mu_{i\,t}Y_{i\,t}=C+\sum_{\Delta =-12}^{\Delta =8}\gamma^{\prime }\,D_{i,t_{0}+\Delta }+\eta_{t}+\varepsilon_{i}+\mu_{i\,t}$$

In Formula (3), *D*_i,t_0+Δ_ refers to the time dummy variable, covering 12 years before and 8 years after policy implementation. The reason for this was that the time span of the samples in this study was 2005–2017, the issuance time of policy of the first area in the experimental group was 2008+1, and the issuance time of policy of the last area in the experimental group was 2017. In addition, the coefficient of the time dummy variable γ’ refers to the difference in the raw milk purchase price of the areas where the policy was implemented and that of the control group regions in the Δ (−12 < Δ < 8) year after policy implementation. The criteria for this test were as follows: if γ’ showed a relatively stable variation during the period with Δ < 0, the policy test conformed to the criteria of the parallel trend test. On the contrary, if γ’ showed large fluctuation during the period with Δ < 0, there was a significant difference in the raw milk selling price variation of the experimental group and that of the control group before the implementation of the policy, which did not conform to the criteria of the parallel trend test.

**FIGURE 2:**
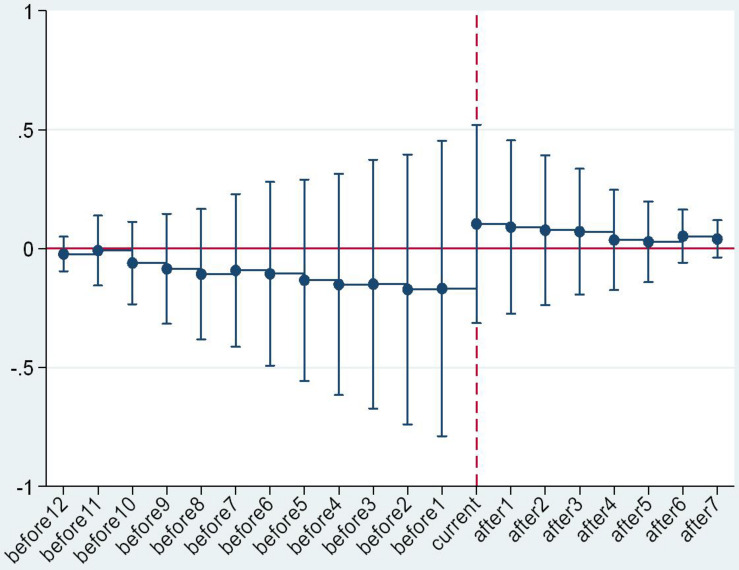
Overall parallel trend test.

**FIGURE 3:**
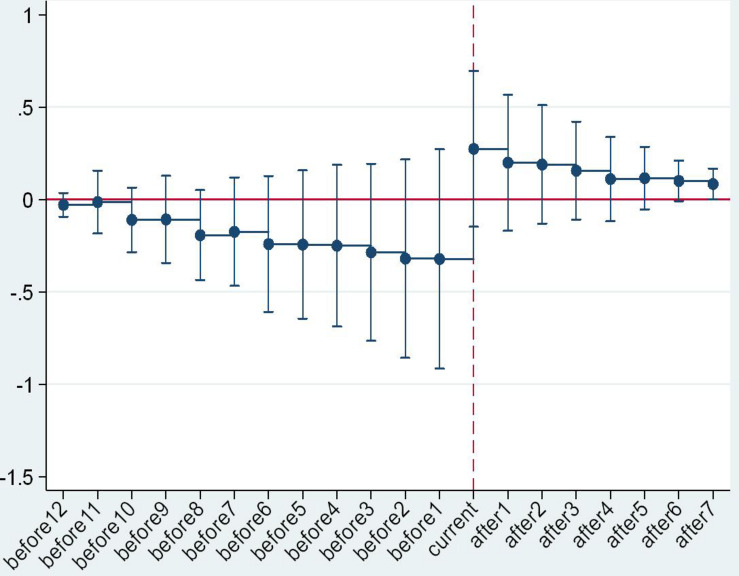
Parallel trend test in the eastern region.

**FIGURE 4:**
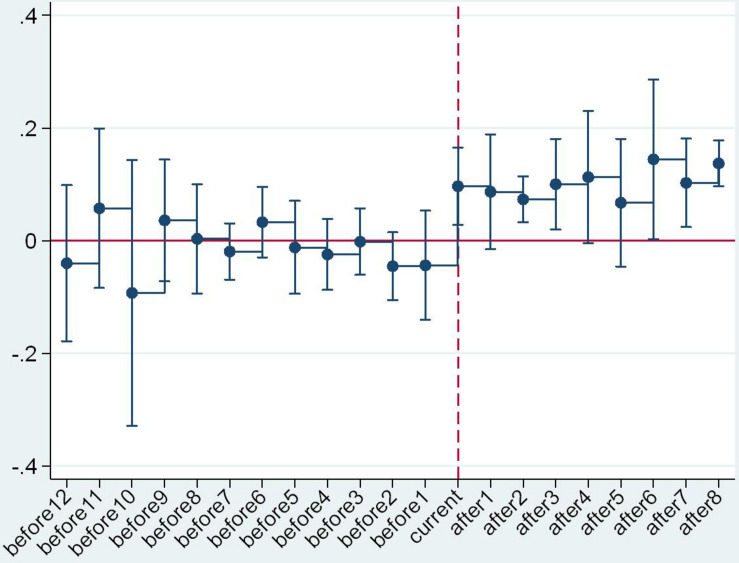
Parallel trend test in the central region.

**FIGURE 5:**
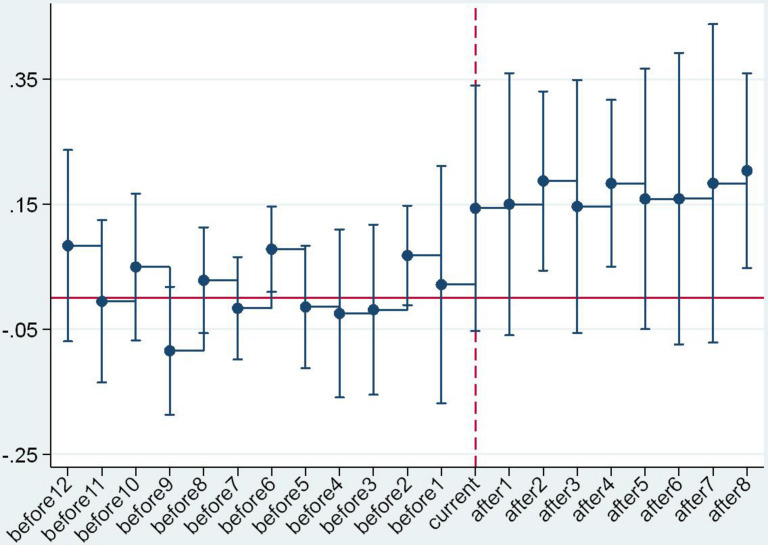
Parallel trend test in the western region.

The parallel trend test results are as shown in Figures 2–5. From a holistic perspective and regional heterogeneous perspective, γ’ showed a relatively stable trend before the issuance time (“current” in the mentioned figures), demonstrating that there was no significant difference in raw milk selling price variation of the areas where the policy is currently implemented and the areas where the policy is not implemented before the issuance of the purchase guide price policy. After the issuance (“current”) time, the value of γ’ increased significantly, showing that the guide price policy had a significant effect on raw milk purchase price. The trend variation of γ’ with the critical value of “current” verified the validity and reliability of the DID results again.

#### Placebo Test

In fact, the parallel trend test was just a necessary precondition for the application of DID. The conformance of the study object to the postulated condition did not mean that the results of the coefficient of the interaction variable reflected the policy effect more than other effects (Guo et al., 2020; Liu et al., 2020; Mao et al., 2020). One important consideration regards the influence of uncontrolled regional time-varying characteristics and undetected factors on estimation results. In this study, all regional characteristic factors that do not vary over time were included by adding a regional fixed effect, and all regional characteristic factors varying over time were included by adding regional time-varying variables, and the regional heterogeneity analysis also showed that regression results were not affected by area selection. However, the time-varying characteristics that were not examined in this study and other undetected factors may have caused bias in results. For this reason, in this study, 19 experimental groups were generated randomly in a fictional way to assess the effect of the guide price policy again, which was repeated 1,000 times, and no significant difference was expected in the experimental group and control group generated randomly.

**FIGURE 6:**
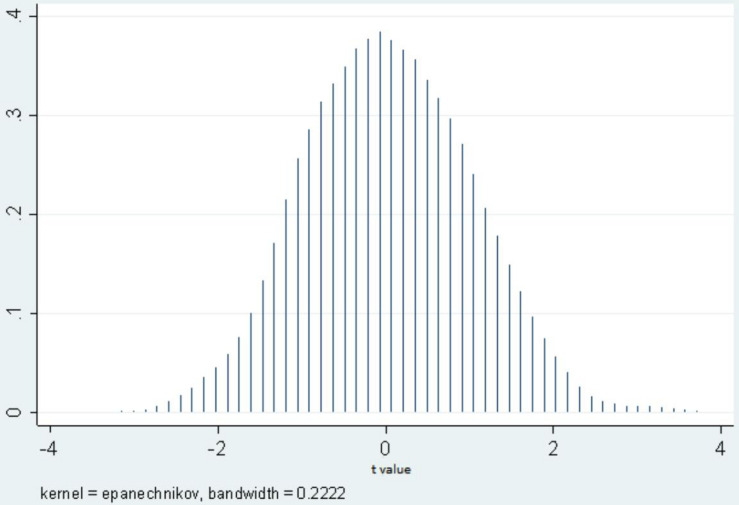
Kernel density estimation of placebo test.

As shown in Figure 6, under the condition of the fictitious experimental group, the t values were distributed in an inverted U-shaped curve with an axis of symmetry of 0, suggesting that the random test results were consistent with expected results, that is, under the condition of experimental groups generated randomly, the guide price policy had no treatment effect on product price in the areas where the policy was implemented. This indirectly indicated that the purchase guide price policy had a positive driving effect on raw milk selling price, which did not change with variation in area and time; this proving a certain robustness.

#### Other Robustness Tests

To further verify the reliability of the research results, a series of robustness tests were conducted based on Formula (2), mainly involving the lag effect and expectation effect of the policy. This was because each area may adopt different expectations and preparations for the purchase guide price policy, and the local market price of raw milk may rise accordingly, generating an expectation effect. In addition, in some cases, the policy may not have a significant influence on the product price of that period, the policy will keep the market in a wait-and-see attitude or in the regulation state, generating a lag effect of the policy. It is certain that the expectation and lag effects can cause bias in conclusions. To avoid the expectation effect or lag effect, in this study, the dummy variables of Lag Phase 1, Lag Phase 2, Expectation Phase 1, and Expectation Phase 2 were added gradually in the model to assess the policy effect again. The results are shown in Table 5. In Table 5, regardless of the lag or expectation effect, the effect of the policy implementation of the current period should be of significance at the 1% confidence level, and the coefficient value should be close to the results shown in Table 3. However, the regression coefficient results of lag and expectation effect were small and not significant, suggesting that the expectation and lag effect of the raw milk purchase guide price policy can be ignored.

**TABLE 5:** Expectation and lag effect test of policy implementation.

Model	12	13	14	15
Period_t_ × Treated_i_	0.1271*** (0.0217)	0.1274*** (0.0220)	0.1098*** (0.0231)	0.1086*** (0.0230)
Period_t_ × Treated_i__–__1_	0.0019 (.0199)	0.0004 (0.0245)	0.0062 (0.0239)	0.0031 (0.0259)
Period_t_ × Treated_i__–__2_		0.0018 (0.0215)	−0.0010 (0.0214)	−0.0001 (0.0235)
Period_t_ × Treated_i__+__1_			0.0088 (0.0172)	0.0101 (0.0191)
Period_t_ × Treated_i__+__2_				−0.0014 (0.0177)
Time fixed effect	Yes	Yes	Yes	Yes
Regional fixed effect	Yes	Yes	Yes	Yes
Weather control variables	Yes	Yes	Yes	Yes
Regional (time-varying) control variables	Yes	Yes	Yes	Yes
Obs.	348	319	290	261
R2	0.4156	0.3772	0.3573	0.3500

*** indicate significance at the 1% levels, respectively, the values in parentheses indicate standard errors. Weather control variables include temperature and precipitation. Regional (time-varying) control variables include per capita GDP and population.

## Further Analysis

The balanced profit distribution among dairy farmers, dairy enterprises, retailers, and consumers is an important precondition for the sustainable development of the dairy industry (Kurata and Ohe, 2020). In other words, although the direct purpose of the raw milk purchase guide price policy is to protect the benefits of dairy farmers, it should fundamentally promote balanced profit distribution in the whole dairy supply chain. The foregoing contents showed that the purchase guide price policy had a positive driving effect on raw milk selling price, that is, the principal purpose (protecting the minimum guaranteed profit of dairy farmers) of the policy was achieved. However, has the profit distribution along the domestic dairy supply chain become more balanced with an increase in the profit of dairy farmers? It is difficult to obtain an intuitive, comprehensive answer by conducting tests only from the producers’ perspective: the test on the policy effect should be conducted from the perspective of processing enterprises and consumers.

**FIGURE 7:**
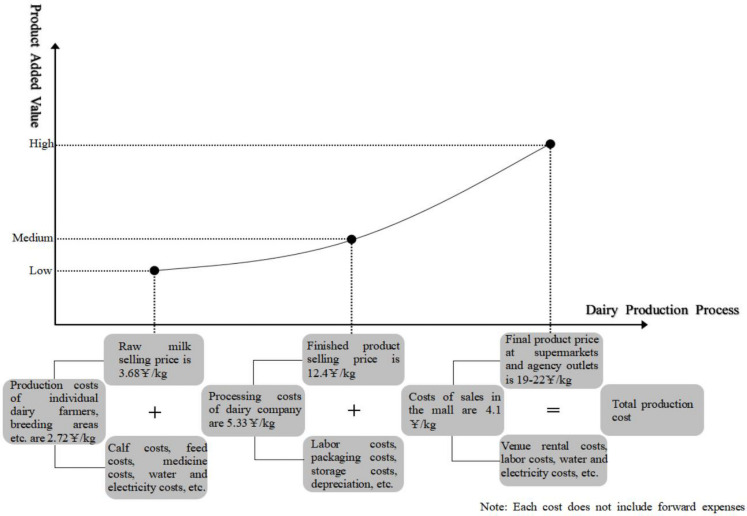
Dairy production cost breakdown.

Prior to the analysis, it is necessary to clearly specify the kind of profit distribution that is deemed relatively reasonable and balanced. In this paper, the research results of Latruffe et al. (2017) were referenced. A Shapley-value approach was adopted to calculate a relatively reasonable profit distribution scheme, according to the contributions of each subject of the chain. The results showed that the balanced profit distribution ratio among dairy farmers, dairy processing enterprises, and retailers should have been 24.5: 23.7: 51.8. However, the actual profit distribution ratio was 10: 35: 55, calculated by using the average price indexes of production, processing, and selling link of dairy products in 2005–2013, as shown in Figure 7. This suggested that the profit in the raw milk supply link was far lower than the reasonable value, and the profit in the processing link and retail link was far higher than the reasonable value. The raw milk purchase guide price policy has increased the selling price of raw milk, resulting in an increase in the profit of dairy farmers; however, has the overall equilibrium level of the supply chain increased? In the following part of this paper, the test and analysis will be conducted using the “ex-factory price index of finished dairy product” and “retail price index of dairy commodities.”

Table 6 shows the effect of the guide price policy on the intermediate processing link and terminal retail link from a holistic perspective, where Models 16 and 17 represent the effect of the raw milk purchase guide price policy on dairy processing enterprises. Models 18 and 19 represent the effect of the policy on retailers. Specifically, the implementation of local raw milk purchase guide price policies had an insignificant inhibiting effect on the product selling price of dairy processing enterprises, which was manifested as a decrease in the finished dairy product price by 0.32% of the unit caused by the policy implementation. On the contrary, the issuance of the guide price policy made the selling price of products in supermarkets and sales commission agencies increase, although the effect was manifested as an increase of 0.11%. Why did the policy have a driving effect on the retailers’ product selling price and the opposite effect on the product selling price of processing enterprises? The possible reason is that, on the one hand, some dairy processing enterprises have established supporting farms, and the price support policy decreased the breeding cost, resulting in a decrease in the selling price of finished dairy products. On the other hand, the price support policy increased the number of cows, resulting in an excessive supply of finished dairy products, thus decreasing the price. However, for retailers, the price support policy could release a market signal, and to prevent the shifting of the production cost of dairy enterprises to themselves, the terminal subjects will reduce profit loss by lifting the price.

**TABLE 6:** Analysis of policy effects from the perspective of milk processing enterprises and retailers.

Model	16	17	18	19
Period_t_ × Treated_i_	−0.0047 (0.0104)	−0.0032* (0.0011)	0.0003 (0.0084)	0.0011* (0.0003)
Time fixed effect	Yes	Yes	Yes	Yes
Regional fixed effect	Yes	Yes	Yes	Yes
Weather control variables	No	Yes	No	Yes
Regional (time-varying) control variables	No	Yes	No	Yes
Obs.	377	377	377	377
R2	0.5088	0.5135	0.8274	0.8292

* indicate significance at the 10% levels, respectively, the values in parentheses indicate standard errors. Weather control variables include temperature and precipitation. Regional (time-varying) control variables include per capita GDP and population.

## Discussion and Conclusion

This paper investigated the effect of the raw milk purchase guide price policy, including the effect on the product selling price of dairy farmers, dairy processing enterprises, and retailers. The effect of the price support policy on the balance of profit distribution in the dairy supply chain was calculated: first, based on the existing profit distribution ratio of 10: 35: 55 among the subjects of the dairy industry, the policy effect was integrated into the ratio and brought through a normalization process to obtain the final profit distribution ratio of 11.35: 34.20: 54.45, which is closer to the balanced profit distribution ratio of 24.5: 23.7: 51.8 than the ratio of 10: 35: 55. The results suggested that the raw milk purchase guide price policy could not only promote an increase in the income of dairy farmers, but also promote a balanced profit distribution among subjects of the dairy supply chain, thus promoting the healthy, stable development of the entire dairy supply chain.

The subjective aim of the dairy product purchase guide price policy is to protect the minimum guaranteed profit of dairy farmers and promote a balanced profit distribution in the dairy industry. This will enhance the production confidence of Chinese raw milk producers and increase Chinese consumers’ expectations and consumer psychology for domestic dairy products. Under the circumstance that production costs remain the same, producers will be more able to resist external risks and more confident in increasing production and R&D investment while improving product quality. Enriching product categories while improving product quality will strengthen consumer confidence in domestic products. In other words, the fundamental goal of the price support policy is to solve the problem of unfair transactions in the raw milk market and increase the production confidence of dairy farmers. Furthermore, the increase in producers’ confidence is conducive to the improvement of product quality, which helps consumers overcome psychological barriers to resisting domestic dairy products and stimulating their consumption vitality. Our study proved that the price support policy is beneficial to improving the production psychology of raw milk suppliers and consumer psychology of consumers.

What then is the actual effect? In this paper, the DID was adopted to test the effect of the price support policy, and the research conclusions include three parts. First, the price support policy generally ensures a minimal guaranteed income of farmers through setting a minimum purchase price, and the actual effect is that the raw milk selling price in the areas where the policy is implemented is 13.54% higher than that in the areas where such a policy is not implemented. The price driving effect is most significant in the western region (15.5%), followed by the eastern region (13%), and the central region (10.73%). Second, the price support policy has an opposite effect on dairy processing enterprises and retailers, although the effect is not obvious. Third, by integrating the effect of the price support policy on subjects of the dairy industry into the current actual profit ratio of the dairy supply chain, the raw milk purchase guide price policy could promote a reasonable revolution in profit ratio among subjects of the chain.

The implications of the policy discussed in this paper are as follows: first, the raw milk purchase price is determined by dairy enterprises, dairy farmers, and industry associations, through negotiation and under the leadership of governments, and is conducive to standardizing the transactions among the subjects of the dairy industry and to establishing a fair and reasonable market structure of raw milk, and thus to protect the income of upstream dairy farmers. This suggests that it is necessary to set it using sustainable institutional regulations. Second, the price support policy may promote sustainable cooperation among the nodes constituting the whole dairy supply chain, and its internal mechanism is giving full play to advantages and bond functions of policy and coordinating profit distribution among subjects in the upstream and downstream portions of the chain. Third, based on the results of the empirical test, it can be derived that, to standardize the market structure of raw milk, it is necessary to handle and publish behaviors not complying with raw milk purchase and selling contracts, creating a list of dishonest dairy processing enterprises, establishing a forced exit mechanism, and strengthening the supervisions by public opinion, thus improving the stability of dairy production, processing, and selling. Scientific and comprehensive evaluation of the raw milk purchase guide price policy can, in the short term, provide theoretical support for the formulation, adjustment, and implementation of relevant policies. In the long term, this will ensure the sustainable development of the dairy supply chain.

## Data Availability Statement

The original contributions presented in the study are included in the article/supplementary material, further inquiries can be directed to the corresponding author/s.

## Author Contributions

All authors undertook research, writing, and review tasks throughout this study, read and agreed to the published version of the manuscript.

## Conflict of Interest

The authors declare that the research was conducted in the absence of any commercial or financial relationships that could be construed as a potential conflict of interest.

## Footnotes

1 Data source: *China Dairy Industry Quality Report* (www.dac.org.cn)

2 Data source: Annual statistics of *Dairy Information Website* on http://www.ryzxw.com/Data/article/list-105-1.html

3 Data source: Annual statistics of *Dairy Information Website* on http://www.ryzxw.com/Data/article/list-105-1.html

4 Data source: http://data.cnki.net/YearData/Analysis

5 Data source: http://data.cnki.net/yearbook/Single/N2018110002

6 Data source: http://data.cnki.net/YearData/Analysis

7 Data source: http://data.cnki.net/yearbook/Single/N2018060291

## References

[B1] Abadie A. (2005). Semiparametric Difference-in-Difference estimators. *Rev. Econ. Stud.* 72 1–19. 10.1111/0034-6527.00321

[B2] Alvarez A. Arias C. (2004). Technical efficiency and farm size: a conditional analysis. *Agric. Econ.* 30 241–250. 10.1111/j.1574-0862.2004.tb00192.x

[B3] Ashenfelter O. (1978). Estimating the effect of training programs on earnings. *Rev. Econ. Stat.* 60 47–57. 10.2307/1924332

[B4] Bogadóttir R. (2020). The social metabolism of quiet sustainability in the faroe Islands. *Sustainability* 12:735. 10.3390/su12020735

[B5] Bryant C. Dillard C. (2019). The impact of framing on acceptance of cultured meat. *Front. Nutr.* 6:103. 10.3389/fnut.2019.00103 31334244 PMC6616100

[B6] Carmen S. S. Patricia R. R. Marta L. P. R. (2016). How can we improve patient satisfaction as a consumer of public health services? The case of psychiatric patients undergoing electroconvulsive therapy. *Front. Psychol.* 7:801. 10.3389/fpsyg.2016.00801 27303355 PMC4880794

[B7] Dolin C. D. Gross R. S. Deierlein A. L. Berube L. T. Katzow M. Yaghoubian Y. (2020). Predictors of gestational weight gain in a low-income hispanic population: sociodemographic characteristics, health Behaviors, and psychosocial stressors[J]. *Int. J. Environ. Res. Public Health* 17:352. 10.3390/ijerph17010352 31947951 PMC6981933

[B8] Dutta R. Pashak T. J. Mccullough J. D. Weaver J. S. Heron M. R. (2019). From consumers to producers: three phases in the research journey with undergraduates at a regional university. *Front. Psychol.* 9:2770. 10.3389/fpsyg.2018.02770 30705658 PMC6344380

[B9] Grafova I. B. Freedman V. A. Lurie N. Kumar R. Rogowski J. (2014). The difference-in-difference method: assessing the selection bias in the effects of neighborhood environment on health. *Econ. Hum. Biol.* 13 20–33. 10.1016/j.ehb.2013.03.007 23623818 PMC4230701

[B10] Guo S. Wang Y. Hou H. Wu C. Yang J. He W. (2020). Natural capital evolution and driving forces in energy-rich and ecologically fragile regions: a case study of Ningxia province. *China*. *Sustainability* 12:562. 10.3390/su12020562

[B11] Hu F. Huang D. F. Xiang R. Xi X. (2020). Research on the export and potential of Chinese dairy products: evidence from 35 countries along the Belt and Road Initiative. *J. Agric. Econ.* 5 130–142.

[B12] Hu F. Ren Z. M. Yu R. J. Huang D. F. Xi X. (2019). Study on the efficiency and influencing factors of domestic dairy farming technology. *Chinese J. Anim. Sci.* 55 139–145.

[B13] Jimenez-Delgado F. Reina-Paz M. D. (2020). Consumer experience and omnichannel behavior in various sales atmospheres. *Front. Sociol.* 11:1972. 10.3389/fpsyg.2020.01972 PMC7427578 32849155

[B14] Khan A. R. Goldringer I. Thomas M. (2020). Management practices and breeding history of varieties strongly determine the fine genetic structure of crop populations: a case study based on european wheat populations. *Sustainability* 12:613. 10.3390/su12020613

[B15] Kniffin K. M. Reeves-Ellington R. Wilson D. S. (2018). When everyone wins? Exploring employee and customer preferences for No-haggle pricing. *Front. Psychol.* 9:1555. 10.3389/fpsyg.2018.01555 30237775 PMC6136271

[B16] Kurata S. Ohe Y. (2020). Competitive structure of accommodations in a traditional Japanese hot springs tourism area. *Sustainability* 12:3062. 10.3390/su12073062

[B17] Latruffe L. Bravoureta B. E. Carpentier A. Desjeux Y. Moreira V. H. (2017). Subsidies and technical efficiency in agriculture: evidence from european dairy farms. *Am. J. Agr. Econ.* 99 783–799. 10.1093/ajae/aaw077

[B18] Liu W. Fan X. Ji R. Jiang Y. (2020). Perceived community support, users’ interactions, and value co-creation in online health community: the moderating effect of social exclusion. *Int. J. Environ. Res. Public Health* 17:204. 10.3390/ijerph17010204 31892188 PMC6982128

[B19] Mao M. Zhang X. Shao Y. Yin Y. (2020). Spatiotemporal variations and factors of air quality in urban central China during 2013–2015. *Int. J. Environ. Res. Public Health* 17:229. 10.3390/ijerph17010229 31905623 PMC6981523

[B20] Qian G. Guo X. Guo J. Wu J. (2011). China’s dairy crisis: impacts, causes and policy implications for a sustainable dairy industry. *Int. J. Sust. Dev. World* 18 434–441. 10.1080/13504509.2011.581710

[B21] Sánchez-López A. M. Menor-Rodríguez M. J. Sánchez-García J. C. Aguilar-Cordero M. J. (2020). Play as a method to reduce overweight and obesity in children: an RCT. *Int. J. Environ. Res. Public Health* 17:346. 10.3390/ijerph17010346 31947884 PMC6981949

[B22] Schmitz A. Helmberger P. (1970). Factor mobility and international trade: the case of complementarity. *Am. Econ. Rev.* 60 761–767.

[B23] Silva R. R. Nina C. Eryn N. Schwarz N. Topolinski S. (2017). Make it short and easy: username complexity determines trustworthiness above and beyond objective reputation. *Front. Psychol.* 8:2200. 10.3389/fpsyg.2017.02200 29312062 PMC5742175

[B24] Tinbergen J. (1964). Shaping the world economy: suggestions for an international economic policy. *Am. J. Agr. Econ.* 46 271–273. 10.2307/1236502

[B25] Wijaya N. Nitivattananon V. Shrestha R. P. Kim S. M. (2020). Drivers and benefits of integrating climate adaptation measures into urban development: experience from coastal cities of Indonesia. *Sustainability* 12:750. 10.3390/su12020750

[B26] Zhang Z. Xu D. Ostrosi E. Cheng H. (2020). Optimization of the product–service system configuration based on a multilayer network. *Sustainability* 12:746. 10.3390/su12020746

[B27] Zhao X. Chi C. Gao X. Duan Y. He W. (2020). Study on the livelihood vulnerability and compensation standard of employees in relocation enterprises: a case of chemical enterprises in the Yangtze River basin. *Int. J. Environ. Res. Public Health* 17:363. 10.3390/ijerph17010363 31948098 PMC6981371

[B28] Zhong Z. Chen S. Kong X. Megan T. (2014). Why improving agrifood quality is difficult in China: evidence from dairy industry. *China Econ. Rev.* 31 74–83. 10.1016/j.chieco.2014.08.008

